# In Vitro Bioactivity of Astaxanthin and Peptides from Hydrolisates of Shrimp (*Parapenaeus longirostris*) By-Products: From the Extraction Process to Biological Effect Evaluation, as Pilot Actions for the Strategy “From Waste to Profit”

**DOI:** 10.3390/md19040216

**Published:** 2021-04-13

**Authors:** Concetta Maria Messina, Simona Manuguerra, Rosaria Arena, Giuseppe Renda, Giovanna Ficano, Mariano Randazzo, Stefano Fricano, Saloua Sadok, Andrea Santulli

**Affiliations:** 1 Dipartimento di Scienze della terra e del Mare DiSTeM, Laboratorio di Biochimica Marina ed Ecotossicologia, Università degli Studi di Palermo, Via G. Barlotta 4, 91100 Trapani, Italy; simona.manuguerra@unipa.it (S.M.); rosaria.arena@unipa.it (R.A.); giuseppe.renda02@unipa.it (G.R.); giovanna.ficano@unipa.it (G.F.); andrea.santulli@unipa.it (A.S.); 2 Istituto di Biologia Marina, Consorzio Universitario della Provincia di Trapani, Via G. Barlotta 4, 91100 Trapani, Italy; mariano.randazzo@tin.it; 3 Dipartimento di Science Economiche, Aziendali e Statistiche, DSEAS, Università degli Studi di Palermo, Viale delle Scienze, Edificio 13, 90100 Palermo, Italy; stefano.fricano@unipa.it; 4 Laboratory of Blue Biotechnology & Aquatic Bioproducts (B3Aqua), Institut National des Sciences et Technologies de la Mer (INSTM), Annexe La Goulette Port de Pêche, La Goulette 2060, Tunisia; salwa.sadok@instm.rnrt.tn

**Keywords:** shrimp by-products, supercritical fluid extraction, astaxanthin, fish oil, SPD, PUFA, proteolytic enzymes, protein hydrolysates, antioxidant activity

## Abstract

Non-edible parts of crustaceans could be a rich source of valuable bioactive compounds such as the carotenoid astaxanthin and peptides, which have well-recognized beneficial effects. These compounds are widely used in nutraceuticals and pharmaceuticals, and their market is rapidly growing, suggesting the need to find alternative sources. The aim of this work was to set up a pilot-scale protocol for the reutilization of by-products of processed shrimp, in order to address the utilization of this valuable biomass for nutraceutical and pharmaceuticals application, through the extraction of astaxanthin-enriched oil and antioxidant-rich protein hydrolysates. Astaxanthin (AST) was obtained using “green extraction methods,” such as using fish oil and different fatty acid ethyl esters as solvents and through supercritical fluid extraction (SFE), whereas bioactive peptides were obtained by protease hydrolysis. Both astaxanthin and bioactive peptides exhibited bioactive properties in vitro in cellular model systems, such as antioxidant and angiotensin I converting enzyme (ACE) inhibitory activities (IA). The results show higher astaxanthin yields in ethyl esters fatty acids (TFA) extraction and significant enrichment by short-path distillation (SPD) up to 114.80 ± 1.23 µg/mL. Peptide fractions of <3 kDa and 3–5 kDa exhibited greater antioxidant activity while the fraction 5–10 kDa exhibited a better ACE-IA. Lower-molecular-weight bioactive peptides and astaxanthin extracted using supercritical fluids showed protective effects against oxidative damage in 142BR and in 3T3 cell lines. These results suggest that “green” extraction methods allow us to obtain high-quality bioactive compounds from large volumes of shrimp waste for nutraceutical and pharmaceutical applications.

## 1. Introduction

The global demand for fish and marine ingredients is growing rapidly, highlighting the need for sustainable management of marine resources, with actions aimed to better understand the intrinsic biodiversity of the marine environment and to preserve it [1]. The recovery of fishery discards, as well as processing waste and marine by-products [2], is necessary so as to save biological resources and to apply the circular economy principle “waste to profit.” This principle consists in a commitment from fishery, aquaculture, and fish processing value chains to develop high-value bio-based marine products with a reduced environmental footprint and to contribute to the United Nations’ Sustainable Developmental Goals.

Among the wide range of marine bioactive compounds, antioxidant carotenoids are a large group of organic and lipophilic pigments, known for their biological activities, which are produced by plants, algae, various bacteria, and fungi, and are present in huge amounts in crustacean wastes [1,3,4,5]. Several studies have been conducted on the beneficial effects of carotenoids in the prevention and management of a large number of diseases, including cancer, cardiovascular diseases, diabetes, osteoporosis, ophthalmic diseases, Alzheimer’s disease, and infectious diseases [3,4,5]. They can also be used as nutritional supplements in nutraceuticals and pharmaceuticals [5,6].

Astaxanthin (AST), contained in the exoskeleton and cephalothorax of crustaceans, is a cheto-carotenoid (3,3′-dihydroxy-β, β-carotene-4,4′-dione) belonging to the family of xanthophylls, derived from the oxidation of β-carotene. Due to its peculiar molecular structure, it has antioxidant, anti-tumor, anti-inflammatory, anti-diabetic, immunomodulatory, and neuroprotective effects [5,6,7,8,9,10,11] that suggest important applications in functional foods, cosmetics, and the food industry [12,13].

This pigment shows higher antioxidant activity than other carotenoids such as α and β-carotene, lutein, lycopene, canthaxanthin, and vitamin E [9]. Due to its cytoprotective and antioxidant capacity, AST has been presented as a promising therapeutic strategy in various ocular diseases [4].

In particular, it has been suggested that carotenoids prevent, delay, and improve retinopathy in diabetes [5]. Baccouche et al. [14] studied the effect of AST, extracted from shrimp waste, on adult retinal cells of the type-2 diabetic model *Psammomys obesus* in hyperglycemic conditions. Their results revealed that AST decreased cell apoptosis, improved mitochondrial function, and improved neurons and the viability of glial cells. Other studies reported that doses of 3 mg/kg, over an eight-week treatment period, reduced retinal oxidative stress and inflammatory mediators in rats with streptozotocin-induced diabetes [15]. The utilization of AST, as a promising therapeutic strategy in ocular disease and in particular in diabetic retinopathy, requires the use of AST extracted using “green” methods, as an alternative to traditional chemical methods that use toxic solvents [16]. The “green techniques” are based on the discovery and design of extraction processes that reduce energy consumption, allow the use of alternative solvents and renewable natural products, and ensure a safe and high-quality extract/product [17,18].

It has been reported that carotenoids can be extracted from shrimp waste using various vegetable oils and their methyl esters (ME) [16,18,19,20,21] or cod liver oil [22]. Sunflower oil and its ME have been recently indicated as potential “green” solvents that could replace traditional organic solvents [18,23]. Extraction using sunflower oil methyl ester was the most efficient “green process” studied in terms of production rate and unit cost of concentrated AST [16,19,20,21]. The use of fish oil, in addition to the advantages already listed regarding the use of vegetable oils and their esters, including excellent solvent properties for the extraction of carotenoids, adds a high content of ω-3 fatty acids [22], which, as reported in the literature, showed, similarly to AST, a beneficial effect in the protection against diabetic retinopathy in obese mice [24].

The “green extraction” of AST could also be performed using supercritical CO_2_ extraction [9,11,16,25,26], which is characterized by high solvent power and selectivity of extraction combined with non-toxicity [9,27,28]; additionally, it is a non-flammable solvent capable of extracting thermolabile compounds. From a sustainability point of view, for the nutraceutical industry the extraction of AST from fresh waste (which also involves high disposal costs) represents a great advantage over its chemical synthesis [9].

Shrimp waste, which is mainly made up of the exoskeleton and cephalothorax, makes up from 50 to 70% of its total fresh weight and could contain other components of high biological value apart from AST, such as bioactive peptides obtained from protein hydrolysates, chitin, and chitosan, whose quantities depend on the species and processing conditions [1,13,29,30,31]. Protein hydrolysates, obtained from shrimp waste by enzymatic reaction, can be integrated in formulated diets for aquaculture as sources of biologically active peptides with a considerable potential in pharmacology and nutraceutic applications and therapies [13,31,32,33,34]. The activities reported in the literature for bioactive peptides are very diverse, ranging from antioxidant power to the measurement of angiotensin-converting enzyme inhibition activity (ACE-IA), which is related to the conversion of angiotensin I into angiotensin II (with beneficial side effects on the control of the hypertension), as well as anti-coagulant activity and the regulation of calcium absorption and immune responses [31,34,35].

*Parapenaeus longirostris* is one of the most important commercial shrimp species, distributed and processed throughout the Mediterranean [29], generating a significant amounts of by-products (BP), that could represent an important source of AST and bioactive peptides, useful for nutraceuticals and pharmaceuticals application.

The aim of the present study was to define a pilot protocol for the green extraction of AST and bioactive peptides from *P. longirostris* BP, demonstrating their bioactivity, in order to reach the zero waste goal, by addressing this important biological resource to other applications, avoiding its waste.

On the basis of reported experiences [16,19,20,21] “green procedures” for AST extraction from large amounts of BP, were represented by supercritical fluid extraction (SFE), fish oils, and ethyl esters (ES). The latter solvent allowed to concentrate AST using short-path distillation (SPD) [16,36] and CO_2_ SFE was also tested as an alternative “green extraction procedure” [9,11,16,25,26]. Finally, AST extracts and bioactive peptides were tested in vitro for antioxidant capacity and ACE-IA. The definition of the pilot scale protocol for the extraction of AST and PH, together with the assessment of its bioactive properties, was addressed to support the value-chains of the *P. longirostris* processing plants to exploit its BP at industrial scale, in order to turn wastes to profit, both for the environment and for the economy.

## 2. Results and Discussion

### 2.1. Proximate Composition and Fatty Acid Profile of P. longirostris By-Product

The proximate composition of BP (exoskeleton including cephalothorax and abdominal parts), reported in Table 1, fall within the ranges reported in the literature, which showed a large variability according to the species [13,37,38,39]. Arbia et al. [38], analyzing the crude exoskeleton composition of *P. longirostris*, reported lower values of ash (25%) and protein (29%) and higher values of chitin (27%) and lipids (15%).

Considering the possible effects of thermal treatment on fatty acid composition, this parameter was evaluated on both wet (WBP) and dry *P. longirostris* by-products (DBP). It is known, in fact, that high temperatures, during the drying phase, can alter the fatty acid profile, leading to a reduction in PUFA content [40,41,42].

In WBP the predominant class was PUFA of n-3 series with a percentage of 36.08 ± 2.94% (Table 2). Within this series, docosahexaenoic acid, DHA (22:6 n3), showed the highest percentage value (21.66 ± 1.73%), followed by eicosapentaenoic acid, EPA (20:5n3) (11.97 ± 1.07%) (Table 2). DBP showed values of 16.45 ± 0.90 and 9.69 ± 0.43% for DHA and EPA, respectively (Table 2). The high content of n-3 fatty acids, especially EPA and DHA, suggests the potential use of these matrices as a source of n-3 [37].

### 2.2. Enzymatic Hydrolysis

**Figure 1:**
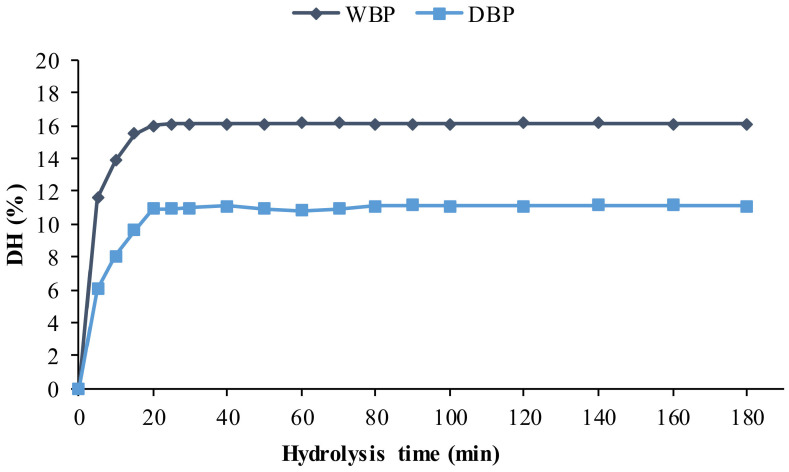
Degree of hydrolysis (DH%) determined in WBP and DBP during reaction with Protamex^®^.

The hydrolysis degree (DH) obtained by Protamex^®^, during the production of protein hydrolysates (PH), from dry and wet *P. longirostris* BP, is shown in Figure 1.

The maximum value of DH (16.07 ± 1.60%) was obtained in WBP after 25 min of reaction, and this value remained constant till the end of the monitoring of the reaction (Figure 1). In DBP the best result was obtained after 20 min of reaction (10.90 ± 1.12%). Results concerning this aspect are diverse and variable in the literature: Dumay et al. [43] reported lower DH using Protamex^®^ and Alcalase^®^ (3.1 and 3.3%, respectively) on viscera of *Sardina pilchardus,* while the 15% DH was obtained, after 15 min of reaction, in by-products of *Xiphopenaeus kroyeri* [13], with the enzyme Alcalase^®^.

### 2.3. Hydrolysates Characterization and Bioactive Properties of the Protein Fractions

#### 2.3.1. SDS PAGE

Sodium dodecyl sulphate poly-acrilamide-electrophoresis (SDS-PAGE) of PH, obtained with Protamex^®^, showed, in accordance to DH variation, a progressive reduction of the relative molecular mass of proteins, obtained at the different reaction times.

**Figure 2:**
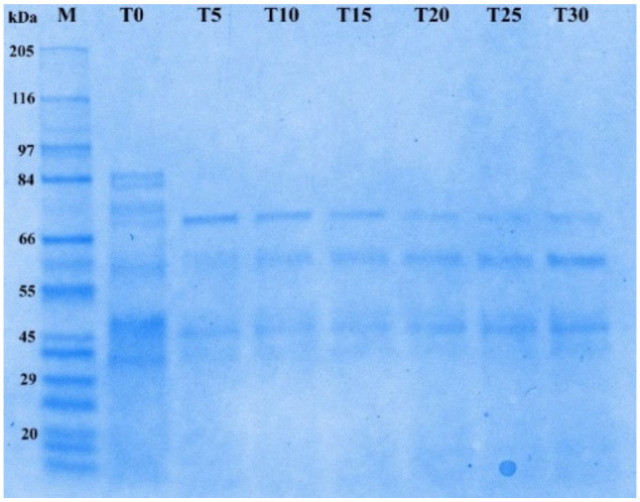
Sodium dodecyl sulphate poly-acrilamide-electrophoresis (SDS PAGE) of *P. longirostris* protein hydrolysates (PH) obtained with Protamex^®^ from T0 to T30 min of reaction. Standard molecular weight marker (M).

In Figure 2, a decreasing intensity of the bands from T5 to T30 can be observed, confirming the hydrolysis of the native proteins. Similar results were obtained by Tkaczewska et al. [44] in *Cyprinus carpio* skin gelatin, attesting the ability of the enzyme Protamex^®^ to produce low-molecular-weight peptides at high DH.

#### 2.3.2. Antioxidant Power of Protein Hydrolysates

**Figure 3:**
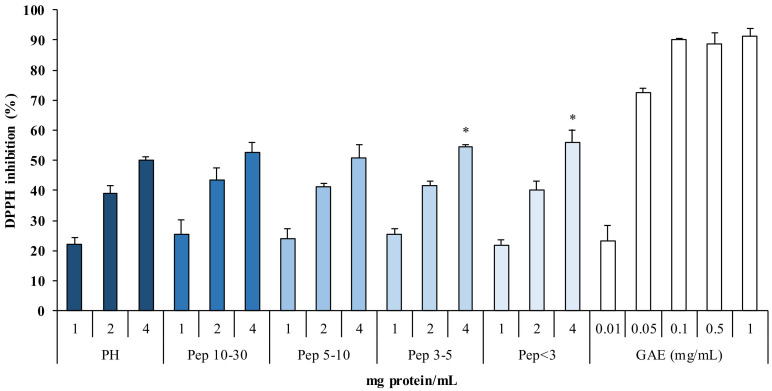
1,1-diphenyl-2-picryhydrazyl (DPPH) radical inhibition (%) of peptide fractions (Pep) at different concentrations (1, 2, and 4 mg protein/mL) obtained by hydrolysis with Protamex^®^. Gallic acid equivalents (GAE 0.01–1 mg/mL) (* *p* < 0.05) compared to activity of PH.

The evaluation of the antioxidant power on peptide fractions (Pep) isolated from PH showed a significant 1,1-diphenyl-2-picryhydrazyl (DPPH) radical inhibition (>50%) at the maximum tested concentration (4 mg protein/mL) (Figure 3).

Showed results indicate that smaller peptides, such as Pep < 3 kDa (55.8% inhibition) and Pep 3–5 kDa (54.5%), had greater antioxidant activity compared to PH.

Similar results were previously reported: protein hydrolysates fractions < 3 kDa from cod, showed the highest antioxidant power in correspondence of the reduction of the peptides size [45]. In the work of Taheri et al. [46], protein hydrolysate fractions, between 10 and 1 kDa, were found to have higher antioxidant power than the fractions at higher molecular weight. This is in accordance with the results of Picot et al. [47], who concluded that the increase in antioxidant power in peptide fractions, compared to PH, may be due to bioactive peptide concentration during the various filtration steps.

#### 2.3.3. ACE-IA Determined by Protein Hydrolysates

**Figure 4:**
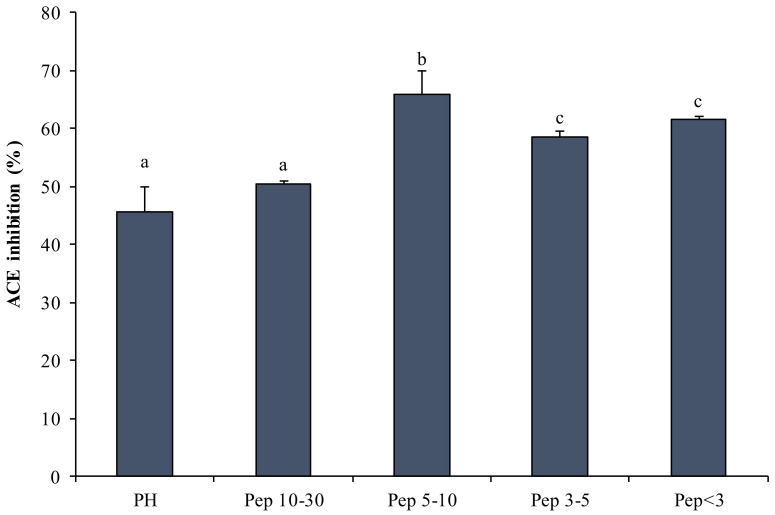
Angiotensin-converting-enzyme inhibition activity (ACE–IA) (expressed in percentage), exerted by peptide fractions obtained by Protamex^®^ and ultrafiltration. Lowercase letters (a–c) indicate significant differences vs. PH (*p* < 0.05).

Figure 4 shows the ACE-IA exhibited by PH. The highest IA was evident in Pep 5–10 kDa (66%) and the lowest among the isolated fractions was evident in Pep 10–30 kDa (50%) (Figure 4).

Figure 4 shows that lower-molecular-weight Pep have higher IA than PH, in accordance with Krichen et al.’s results [48], which indicated a higher activity in peptide fractions from protein hydrolysates of shrimp waste (*P. longirostris*) than in whole hydrolysates.

These results provide further support for the use of PLPD to produce hydrolysates with bioactive peptides.

### 2.4. Extraction, Enrichment and Determination of AST

#### 2.4.1. AST Yields Extracted with Crude Viscera Oil (CVO) and Ethyl Esters

Table 3 shows the AST yields after extraction using CVO, including total fatty acids ethyl esters obtained from CVO (TFA), polyunsaturated fatty acid ethyl esters enriched by SPD (PUFAE), and exhausted fatty acid ethyl esters (EFA) from WBP and DBP, at different extraction ratios (ER: 0.5, 1.0, and 2.0 solvent volume/waste weight). The obtained yields were always higher when increasing the extraction ratio, for both WBP and DBP, reaching the highest values with the 2.0 ratio.

For all extraction ratios with the CVO, TFA, and EFA solvents, WBP showed significantly higher yields (*p* < 0.05) than DBP, whereas no significant differences were observed for PUFAE (Table 2).

Utilizing the extraction ratio of 2.0, in WBP, the higher AST yield was reached with TFA (160.06 ± 8.91 µg/g of dry weight (d.w.)) followed by CVO (149.06 ± 0.82 µg/g d.w.), PUFAE (59.06 ± 4.06 µg/g d.w.), and finally EFA (50.50 ± 0.91 µg/g d.w.) (*p* < 0.05). Consequently, the highest AST concentration was found in the extract obtained from WBP with TFA (20.64 ± 0.40 µg/mL); it was significantly higher than that obtained with the CVO (18.86 ± 0.21 µg/mL), PUFAE (7.67 ± 0.36 µg/mL), and EFA extracts (5.31 ± 0.18 µg/mL) (Table 2).

These results are in accordance with those of Sachindra and Mahendrakar [19], who found that the AST yield of *Penaeus indicus* by-products, extracted using vegetable oils, is higher using an extraction ratio equal to 2.0. Chen and Meyers [49] obtained maximum pigment yield from crawfish by-products incubating the mixture at a temperature of 80–90 °C for 30 min. However, temperatures above 70 °C and times above 150 min significantly reduced AST yields in *P. indicus* [19]. As carotenoids are degraded at higher temperature, Sachindra and Mahendrakar [19] suggest to use lower temperature for longer time to optimize extraction yield of carotenoids from shrimp by-product [19]. Parjikolaei et al. [20] also found better extraction efficiencies in *Pandalus borealis* by-products by applying a temperature of 70 °C, also considering a shorter extraction time.

The obtained results are in agreement to Parjikolaei et al. [20] that using ME from vegetable oil, reported a significantly higher extraction efficiency for a wet matrix compared to a freeze-dried matrix, as freeze-drying leads, to some extent, to the collapse of the solid structure of biological materials [50]. This probably makes the internal freeze-dried matrix less accessible to solvents, reducing both the mass transport rate and the yield. This could explain similar and relatively lower yields of dried samples with respect to wet samples, for all solvents used for freeze-dried matrix extraction (Table 2). In this work, the matrix was dried in a ventilated oven and may have been affected by similar modifications, or AST could have been partially degraded due to its sensitivity to drying temperatures [20]. The yields of WBP obtained using TFA as a solvent were higher than yields obtained utilizing CVO as solvent, in accordance with the results showed by Parjikolaei et al. [20] in *P. borealis* by-products.

Furthermore, similar values were reported by Shahidi and Synowiecki [22], who utilized a dry matrix and cod liver oil as a solvent, with a yield of 147 µg/g in *P. borealis* by-products.

The higher extraction efficiency obtained by Parjikolaei et al. [16,20] using TFA can be explained considering the lower viscosity of ME compared to sunflower oil, as well as the different polarity and interactions between AST, ME, and sunflower oil.

#### 2.4.2. Supercritical CO_2_ Extraction (SFE)

The AST yields obtained by SFE from DBP (4.84 ± 0.06 µg/g) were similar to Messina et al. [11] and to the data reported by Radzali et al. [9], but resulted lower than data reviewed by Ahmadkelayeh and Hawboldt [25].

A low recovery of AST and a low selectivity has been reported in samples extracted using supercritical CO_2_ compared to traditional chemical methods [51]. This problem can be overcome by using appropriate co-solvents, at different concentrations, which could improve yield, solubility, and extraction efficiency [9,16,25] [9,16,25]. Although SFE is considered a green method showing several advantages, such as the use of non-toxic solvents, there is a need to optimize the balance among large-scale extraction processes and investment and operating costs [25]. It is worth stressing that the production costs of AST using sunflower oil or the methyl ester of sunflower oil extraction (0.06 and 0.16 $/mg of AST, respectively) are lower than those using hexane isopropanol as a solvent or SFE (about 0.6 and 0.82 $/mg of AST, respectively) [16].

#### 2.4.3. AST Enrichment by Short Path Distillation (SPD)

**Figure 5:**
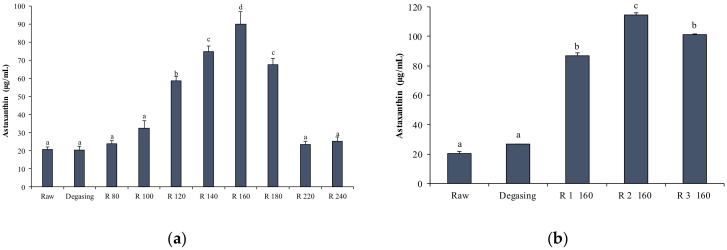
(**a**) AST concentrations (µg/mL) obtained in the residue via short path distillation (SPD) at different evaporation temperatures. (**b**) AST concentrations (µg/mL) obtained in the residue via SPD by repeating several steps at the same evaporation temperature (160 °C). Different letters (a,b,c, …) indicate significant differences (*p* < 0.05).

SPD was applied to enrich AST extracted using TFA. In SPD, at a specific temperature and pressure, lighter molecules evaporate and condense in the distillate, while heavy molecules such as AST are recovered and concentrated in the heavy phase. Using SPD, the maximum concentration was reached at evaporation temperature of 160 °C (89.77 ± 7.12 µg/mL) and pressure of 0.002 mbar, with an overall concentration increase of about 4.5 times compared to the initial concentration (Figure 5a). However, maximum AST concentrations were lower than those reported by Parjikolaei et al. [16,21], who obtained an increase from 3.04 to 155 ppm with an evaporation temperature of 140 °C and a pressure of 1000 Pa. A significant decrease at evaporation temperatures above 160 °C was reported by Batistella et al. [52]. This result was attributed to a degradation of the molecule, as an increase in carotenoid decomposition was obtained at temperatures from 150 to 170 °C [52].

To obtain a further concentration of AST, at the evaporation temperature 160 °C, three steps were repeated each time, recycling the previously enriched heavy fraction. The first two steps (R1 and R2) showed a significant AST enrichment present in the heavy phase, of up to 114.80 ± 1.23 µg/mL. The third step (R3) showed a significant decrease compared to the second step (101.33 ± 0.65), probably due to the degradation of the molecule in the evaporator [52] (Figure 5b).

### 2.5. Evaluation of Bioactive Properties of Protein Hydrolysates and AST In Vitro

#### 2.5.1. Antioxidant Activity of Hydrolyzed Fractions in Human Fibroblast (142BR)

The antioxidant properties of the peptide fractions obtained by protein hydrolysates of BP were tested on a human fibroblasts cell line (142BR) against induced oxidative stress, utilizing previous standardized protocol, which employs hydrogen peroxide as an inducer [11].

**Figure 6:**
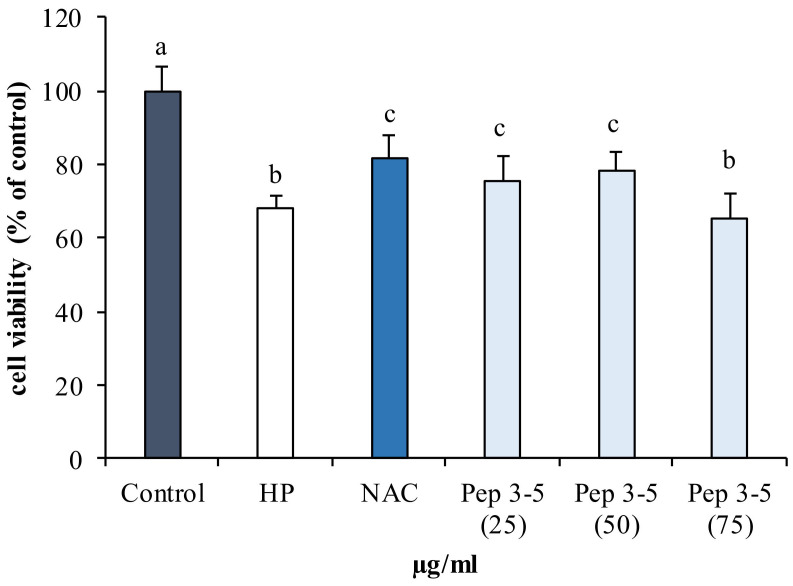
Effect of hydrolyzed fractions on 142RB fibroblast cells exposed to oxidative stress induced by hydrogen peroxide (50 µM). Control–cells maintained in standard culture conditions; HP–hydrogen peroxide treatment; NAC–cells pretreated with the synthetic antioxidant *N* acetylcysteine. Peptide fractions were obtained by ultrafiltration of BP hydrolysates with Protamex^®^ (Pep). Different letters (a, b, c) indicate significant differences (*p* < 0.05).

As expected, the cell viability showed a statistically significant reduction (*p* < 0.05) in cells exposed to hydrogen peroxide (HP) compared to the control (Figure 6).

By contrast, cells pretreated with different concentrations (25–75 µg/mL) of purified peptides (Pep 3–5 kDa) and subsequently exposed to hydrogen peroxide, showed a higher viability than untreated stressed cells (HP), which is comparable to cells treated with the synthetic antioxidant NAC (Figure 6).

The cell viability showed a dose-dependent trend at concentrations of 25 and 50 µg/mL, and then decreased again at 75 µg/mL. Viability was higher at the concentration of 50 µg/mL.

These results suggest that low-molecular-weight peptides have protective effects against oxidative damage, according to the results obtained by Qian et al. [53] in human embryonic lung fibroblasts cell lines (MRC-5) and mouse macrophages cells (RAW264.7). These authors report that low-molecular weight peptides exhibit high scavenger activity. Peptides with a low molecular weight according to Sabeena Farvin et al. [45] may act as hydrogen donors and thus convert free radicals into more stable products. In fact, as described above (Figure 3), smaller peptide such as Pep 3–5 kDa exhibited high antioxidant activity and are able to protect cells from oxidative stress.

These observations confirm that peptides produced via enzymatic hydrolysis from marine resources could represent an alternative source of bioactive compounds with high antioxidant activity that could be used to counteract oxidative stress [54].

#### 2.5.2. In Vitro Effects of AST Extracted Using Fish Oil, Ethyl Esters, and SFE

AST extracted using different “green” solvents was tested on a 3T3 cell line exposed to oxidative stress by the chemical inducer hydrogen peroxide (50 µM).

**Figure 7:**
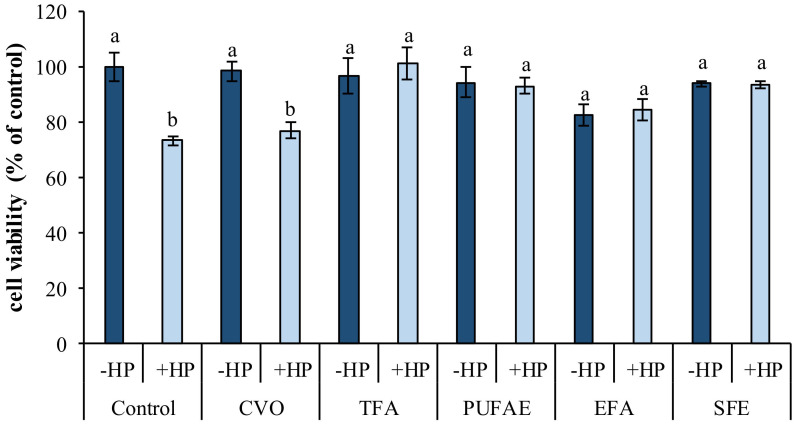
Effect of AST extracted using various methods on the viability of 3T3 cells exposed to oxidative stress with hydrogen peroxide (50 µM). HP-hydrogen peroxide treatment; Control-cells maintained in standard culture conditions; CVO-crude viscera oil; TFA-ethyl esters of total fatty acid obtained from CVO; PUFAE-polyunsaturated fatty acid ethyl esters enriched by SPD; EFA-exhausted fatty acid ethyl esters; SFE-supercritical fluid extract. Different letters (a,b) indicate significant difference (*p* < 0.05).

As expected, the viability underwent a reduction (*p* < 0.05) in cells exposed to hydrogen peroxide alone compared to control cells (Figure 7).

Pre-incubation of cells for 24 h with TFA, PUFAE, and SFE significantly inhibited the cytotoxic effects of the pro-oxidant (*p* < 0.05) (Figure 7).

A further confirmation of the antioxidant power was obtained in a preliminary test conducted in a similar cell line and under the same conditions as in previous work (data not shown) [11].

However, the reduction in viability in cells pretreated with CVO shows that the latter is not able to protect cells from oxidative stress (Figure 7). These results indicate that AST exerts an antioxidant effect on oxidative injuries, the extent of which is also dependent on the solvent used for its extraction and dissolution. Results are in accordance to AST’s neuroprotective effect demonstrated in primary retinal cells against high glucose-induced retinal damage [14] and its cytoprotective role in the human neuroblastoma cell line SH-SY5Y [55].

## 3. Materials and Methods

### 3.1. Sampling and Sample Processing

By-products of *P. longirostris*, composed of cephalothorax and abdominal parts, were sampled from commercial processing plants located in Mazara del Vallo (Trapani, Italy) and Portopalo (Siracusa, Italy), stored in cold containers, transported to the laboratory, and frozen at −25 °C. The preparation of DBP was carried out in 150 kg batches.

WBP was dried in a ventilated thermostat (volume 540 L) at 60 °C for 72 h and ground. The dried matrix was fractionated on a sieve column and stored under vacuum at 4 °C.

### 3.2. Evaluation of Proximate Composition and Fatty Acid Profile of P. longirostris by Products

The analyses for proximate composition and fatty acid profile were performed in two replicates, to determine the moisture content and ash content [56], crude protein [57] and total lipid [58]. Chitin percentage content of was determined by stoichiometric calculation of total nitrogen content [39]. Fatty acids were extracted and transesterified according to Lepage and Roy [59] and determined using gas chromatography [60].

### 3.3. Enzymatic Hydrolysis and AST Extraction

Enzymatic hydrolysis was performed on WBP and DBP in two replicates. The reaction was carried out on 10 Kg of WBP or DBP in distilled water (1:1 water volume/waste weight), in 50 L steel reactors under constant agitation and continuous pH value monitoring. To control the reaction temperature of the mixture with high efficiency, the steel reactor was equipped with an internal helical heating coil and connected to a Julabo SE-6 Heating circulator, temperature range −5/350 °C (Seelbach, Germany) filled with Marlotherm^®^ SH (Global Heat Transfer Ltd., Stone, UK). The bacterial protease Protamex^®^ (>1.5 Anson Units (AU)-*N*/g protein) (Sigma-Aldrich, St. Louis, MO, USA) was used for enzymatic hydrolysis.

Protamex^®^ was added (3% *w*/*w* of WBP or DBP), for 180 min at 60 °C, pH at 8 adjusted by 5 *N* NaOH [13,43]. The degree of hydrolysis (DH%) was determined by direct evaluation, as described by Adler-Nissen [61].

To calculate DH%, the value of 7.7 for h_tot_ was used, as reported by Duarte de Holanda and Netto [13] for enzymatic hydrolysis in *X. kroyeri* by-products. We calculated the dissociation factor (α value) using the equation described by Duarte de Holanda and Netto [13]. Total of 500 mL aliquots of reaction mixture (collected after 5, 10, 15, 20, 25, 30 until 180 min from enzyme addition) were heated to 90 °C in 4 min, and kept at this temperature for 5 min to inactivate enzyme activity [13] in a microwave oven as described by Utne-Palm et al. [62].

AST was extracted from the hydrolysate by different “green” solvents: crude viscera oil (CVO), total fatty acids ethyl esters obtained from CVO (TFA), polyunsaturated fatty acid ethyl esters enriched by SPD (PUFAE) and exhausted fatty acid ethyl esters(EFA), using extraction ratios of 2.0, 1.0, and 0.5 (oil volume/WBP or DBP weight) [19], for 150 min at 70 °C, in a steel reactor, as described by Sachindra and Mahendrakar [19].

The following solvents were added to the hydrolysate in the steel reactor: CVO obtained from sea bream, TFA, PUFAE, and EFA, extracted and enriched as reported by Messina et al. [63].

### 3.4. Separation of Protein Hydrolysates and AST

Extraction mixtures, obtained after hydrolysis reaction, containing an apolar component (AST) and a polar component (protein hydrolysates) were filtered through a 125 µm sieve and centrifuged using a continuous high speed tubular centrifuge (CEPA, Carl Padberg, Zentrifugenbau GmbH, Lahr/Schwarzwald, Germany) at room temperature, feed flux of 15 L/h, at 38,454 g, to contemporary and continuously separate the solids (retained in the cylinder), the aqueous phase (containing protein hydrolysates), and the lipid phase (containing AST).

### 3.5. Protein Hydrolysates Characterization

#### 3.5.1. SDS-PAGE

For evaluation of the relative molecular mass of the proteins present in the samples, both total homogenate and hydrolysates from BP obtained in the various hydrolysis steps (5, 10, 15, 20, 25, and 30 min) were separated using SDS-PAGE. Aliquots of hydrolysates (0.5 mL) were centrifuged at 10,621 g for 1 min, and 200 µL of supernatant was recovered for protein quantification. The concentration of total proteins in all samples was determined using the method of Lowry et al. [64], using BSA (bovine serum albumin) Sigma-Aldrich) as standard.

Aliquots of 100 μg of protein, diluted with a Laemmli buffer (Sigma-Aldrich) and denatured for 5 min at 90 °C, were loaded onto a gradient polyacrylamide minigel (4–15%) (Bio-Rad, Hercules, CA, USA) and subjected to electrophoresis at 20 mA for about 2 h [65]. A mix of standard proteins with relative molecular masses varying between 250 and 14 kDa (Bio-Rad, Hercules, CA, USA) was run simultaneously into the gel. After the electrophoretic run, the gel was stained with Coomassie Blue (GelCode Blue Stain Reagent, Pierce, Rockford, IL, USA). The image of the gel was acquired and elaborated using the software Image Lab 4.1 (Bio-Rad, Hercules CA, USA).

#### 3.5.2. Fractionation of Proteins

PH, obtained with enzymatic Protamex^®^, were filtered on a 0.45 μm nylon membrane filter (Pall, Ann Arbor, MI, USA) to remove the coarsest material. The filtrate PH was then fractionated and concentrated according to molecular weight by ultrafiltration on continuous tangential filtration modules (Vivaflow 200, Sartorius, AG, Germany). The fractionation was carried out, in sequence, by membranes of molecular weight cut-offs (MWCOs) of 30, 10, 5, and 3 kDa [47].

Protein hydrolyzed fractions (PH), having different molecular mass, in relation to the cut-off of the membranes, (Pep 10–30 kDa, Pep 5–10 kDa, Pep 3–5 kDa, Pep < 3 kDa), were frozen at −80 °C and freeze-dried.

The lyophilized hydrolyzed peptides were reconstituted in distilled water at a concentration of 30 mg/mL (stock solution) and the pH was adjusted to 7.0. The concentration of the obtained fractions was determined with the Lowry assay [64].

#### 3.5.3. DPPH Radical Scavenging Activity

The total antioxidant power of PH and Pep was measured using the DPPH assay [66,67,68]. A total of 0.4 mL of different Pep, at concentration of 1, 2, and 4 mg/mL, was prepared by diluting the stock solutions in absolute ethanol (30 mg/mL); they were then mixed with 1.6 mL of 100 μM DPPH in 96% ethanol to start the reaction. The reaction mixture was kept at room temperature and the absorbance was measured at 517 nm after 30 min. Gallic acid (Sigma-Aldrich) was used as a positive control.

Scavenging activity was determined using the following Equation (1):

(1) $$\mathrm{Scavenging}\;\mathrm{activity}\;\left(\%\right)=\;\left[1-\left(\mathrm{Absorbance}\;\mathrm{sample}/\mathrm{Absorbance}\;\mathrm{control}\right)\right]\times 100$$

#### 3.5.4. ACE Inhibition Assay

The ACE inhibitory activity (ACE-IA) of PH and Pep was measured using the Cushman and Cheung method [69], with slight modifications [70]. The sample solution (1 mg protein/mL in 50 mM buffered sodium borate (SBB) pH 8.3, 50 µL) was preincubated with a solution of the angiotensin converting enzyme (ACE, from rabbit lung) (50 µL, 25 U/mL in SBB) (Sigma-Aldrich) at 37 °C for 10 min and then mixed with the enzyme substrate N-ippuryl-l-histidyl-l-leucine (150 µL, 8.3 mM in SBB) for 30 min at the same temperature. The reaction was blocked with HCl 1 M (250 µL). The resulting hippuric acid was extracted from the acidified solution (0.5 mL) of ethyl acetate by vortex mixing for 15 s. After centrifugation (800 g, 15 min), the supernatant (0.2 mL) was dried by evaporation under vacuum conditions for 2 h [70]. The obtained hippuric acid was dissolved in distilled water and the absorbance was measured at 228 nm with a spectrophotometer. ACE inhibitory activity was calculated according to Formula (2):

(2) $$\mathrm{ACE}\;\mathrm{inhibitory}\;\mathrm{activity}\;\left(\%\right)\;=\;\left[\left(B\;-\;A\right)/\left(B\;-\;C\right)\right]\;\times 100$$

where B is the absorbance of the uninhibited control, C is the absorbance of the inhibited reaction, and A is the absorbance in the presence of PH.

### 3.6. AST Supercritical Fluid Extraction (SFE)

The system used for SFE (Helix System Basic Model, Applied Separation, Allentown, PA, USA) is equipped with an extraction vessel and a separator with volumes of 500 mL that can operate at a maximum pressure of 690 bar and a maximum temperature of 160 °C. The extraction parameters and procedures were described by Messina et al. [11]. Briefly, the dried and ground matrix was passed on a sieve column in order to obtain the suitable particle size fraction (250–500 µm) for the extraction. The extraction temperature and pressure were set at 40 °C and 350 bar, respectively. After 30 min of static, the dynamic extraction was carried out with a CO_2_ flow regulated at 2.5 L/min for 2 h.

### 3.7. Enrichment of AST by Short Path Distillation (SPD)

AST samples extracted using TFA as a solvent were processed by SPD. The molecular distiller with the evaporator type “falling film” (model VLK 70-4 FDRR-SKR-T, VTA Gmbh, Niederwinkling, Germany) has an evaporation surface of 4.8 dm^2^, which allows to process up to 1.5 kg h^−1^ of oil at a maximum operating temperature of 350 °C and a vacuum operating pressure of 0.001 mbar [71].

The ethyl ester mixture was loaded into the feed vessel by a peristaltic pump preheated to 40 °C and prepared for a first degassing step to remove impurities and solvent traces from the mixture. Subsequently, several distillation cycles were carried out at increasing evaporation temperatures (80, 100, 120, 140, 160, 180, 220, and 240 °C) on the starting ethyl esters (Table 4).

Aliquots of the enriched fraction (heavy phase) and of the distillate (light phase) were sampled, diluted, and analyzed at the spectrophotometer. A second enrichment test was performed by processing the ethyl esters at the evaporation temperature (160 °C) at which the highest AST concentration was obtained, and three cycles were repeated at the same temperature on the same enriched fraction to obtain a further concentration.

### 3.8. Spectrophotometric Determination of AST

For the determination of AST in the different extracts (CVO, TFA, PUFAE, EFA, and SFE) and enriched fractions (obtained from both WBP and DBP samples), after appropriate dilution, the samples were determined spectrophotometrically at 486 nm applying Equation (3) by Sachindra and Mahendrakar [19] for vegetable oils but considering the molar extinction coefficient $E_{1\mathrm{cm}}^{1\%}$ (2043) calculated by Chen and Myers [72] for fish oil.

(3) $$\mathrm{Carotenoid}\;\mathrm{as}\;\mathrm{AST}\;\left(\mu g/g\;\mathrm{BP}\right)=\frac{A\times V\times D\times 10^{6}}{100\times E_{1\mathrm{cm}}^{1\%}\times W}$$

where *A* is the absorbance at 486 nm, *V* is the volume of pigmented oil recovered, *D* is the dilution factor, *W* is the weight of BP in grams, and $E_{1\mathrm{cm}}^{1\%}$ is the extinction coefficient.

### 3.9. Cell Culture

The antioxidant properties of SFE and AST extracted by utilizing CVO, TFA, PUFAE, and EFA as solvents were determined in two cell lines: human skin fibroblast cell line, 142BR (ECACC n. 90011806, Sigma^®^) and 3T3 L1 cell lines from mice (ECACC n. 86052701, Sigma^®^).

142BR cells were grown in minimum essential medium Eagle (MEME) supplemented with 15% fetal bovine serum (FBS, 2 mM glutamine, 1% non-essential amino acids and 100 µg/mL penicillin–streptomycin). 3T3 cells were cultured in Dulbecco’s modified Eagle’s medium (DMEM), supplemented with 10% calf serum, 2 mM glutamine, and 100 µg/mL penicillin–streptomycin. All reagents were from Sigma-Aldrich.

#### 3.9.1. Assessment of Antioxidant Activity of Pep in 142BR Cells

Confluent cell cultures were trypsinized and seeded in a 96-well plate at a concentration of 1 × 10^4^ cells/well and incubated for 24 h.

Different concentrations of Pep (25, 50, and 75 µg/mL dissolved in sterile distilled water and filtered in 0.22 µm Millipore membrane) (Millex^®^ Merck Millipore, Darmstadt, Germany), were added to cells and incubated for 48 h. After 48 h, all samples except for the control were exposed to the chemical promoter of oxidative stress, hydrogen peroxide (50 µM) (Carlo Erba reagents, Milano, Italy), according to a previously standardized protocol [11,66,68,73,74,75,76]. The viability was measured using the 3-(4,5-dimethyl-2-yl)-2,5-diphenyltetrazolium bromide (MTT) assay, according to Mossman’s method [77], as reported by Messina et al. [11]. The data were expressed as the mean percentage of viable cells as compared to the respective control culture. Each concentration was tested in three replicates, each consisting of five single determinations.

#### 3.9.2. Protective Effect AST Extracted by CVO, TFA, PUFAE, EFA, and SFE in 3T3 Cells

Cells were seeded in a 96-well plate at a concentration of 7 × 10^3^ cells/well and incubated for 24 h. After 24 h the cells were treated with AST extracted by CVO, TFA, PUFAE, EFA, and SFE, dissolved in ethanol, and utilized at a concentration of 0.2 nM in the medium, with a final solvent concentration of 0.1% (*v/v*), and left to incubate for 24 h, as described by Messina et al. [11].

A preliminary toxicity test, aimed to assess the effect of “green solvents” on cell vitality, was carried out in a recent paper, where it was demonstrated that these solvents do not influence cell viability [63].

Then all cells, except for the control, were exposed to hydrogen peroxide (50 µM) as described above, to evaluate the protective effect of AST against oxidative stress.

### 3.10. Statistical Analysis

Statistical analysis was performed using the computer application SPSS for Windows^®^ (version 20.0, SPSS Inc., Chicago, IL, USA). All analyses were carried out in triplicate. The results are expressed as mean ± standard deviation. The homogeneity of variance was confirmed by the Levene test. Data were subjected to one-way analysis of variance (ANOVA), and Student–Newman–Keuls or Games–Howell post-hoc tests were performed in order to make multiple comparisons between experimental groups. The significance level was 95% in all cases (*p* < 0.05).

## 4. Conclusions

The possibility to obtain bioactive compounds, such as AST and bioactive peptides, from the shrimps processing value-chain, could support the goal to increase the sustainability of marine resources, avoiding to waste nutrient-rich marine by-products, and could help also to turn waste into profit for the enterprises.

The “green” AST extraction methods, using CVO, TFA, PUFAE, EFA, and SFE, have shown good yields, especially in samples obtained by TFA as a solvent; the AST can be enriched by SPD.

This study demonstrated that different AST extracts have cytoprotective and antioxidant effects in vitro. Obtained results attest to their possible application in pharmaceutics and nutraceutics.

In view of the reported results on the protective effects against retinopathy of both AST [4,14,78] and fatty acids n-3 [24,78], obtained data are particularly interesting because they allow to obtain a product that contains both AST and fatty acids at the same time, and therefore could ensure, through their synergistic effect, a greater protection against ocular diseases.

Bioactive peptides obtained after enzymatic reaction and ultrafiltration show that Peps reveal an increased scavenger activity and shows inhibitory effects on ACE compared to PH.

In addition, “green” methods, used instead of traditional methods that employ chemical solvents, make it possible to obtain high-quality bioactive compounds from large volumes of BP, for application in the pharmaceutical and nutraceutical sectors.

## Figures and Tables

**Table 1:** Proximate composition (g/100 g DW ^a^) of *P. longirostris* by-products (BP).

	g/100 g
Moisture	3.43 ± 0.16
Ash	36.40 ± 0.61
Lipid	4.96 ± 0.17
Protein	38.47 ± 0.46
Chitin	16.75 ± 1.06

^a^ sample dry weight.

**Table 2:** Fatty acid profile (% of total fatty acids) of wet (WBP) and dry (DBP) *P. longirostris* by-products. EPA: eicosapentaenoic acid; DHA: docosahexaenoic acid.

	WBP	DBP
14:0	1.47 ± 0.06	0.83 ± 0.08
16:0	17.67 ± 0.20	13.10 ± 0.61
16:1n-7	3.40 ± 0.22	2.46 ± 0.13
16:2n-4	0.38 ± 0.05	0.29 ± 0.03
16:3n-4	0.70 ± 0.06	0.67 ± 0.04
18:0	6.61 ± 0.18	6.02 ± 0.13
18:1n-9	18.25 ± 1.91	22.84 ± 0.55
18:1n-7	4.03 ± 0.18	3.81 ± 0.16
18:2n-6	4.00 ± 1.67	15.24 ± 0.51
18:3n-4	0.23 ± 0.05	0.19 ± 0.03
18:3n-3	0.52 ± 0.04	0.36 ± 0.04
18:4n-3	0.29 ± 0.05	0.16 ± 0.02
20:1n-9	1.54 ± 0.07	1.34 ± 0.04
20:4n-6	4.68 ± 0.39	4.32 ± 0.33
20:4n-3	0.38 ± 0.05	0.23 ± 0.06
20:5n-3 (EPA)	11.97 ± 1.07	9.69 ± 0.43
22:1n-11	0.68 ± 0.06	0.53 ± 0.04
22:1n-9	0.31 ± 0.08	0.26 ± 0.06
22:5n-3	1.26 ± 0.27	1.04 ± 0.14
22:6n-3 (DHA)	21.66 ± 1.73	16.45 ± 0.90
Saturated	25.74 ± 0.31	19.95 ± 0.74
Monounsaturated	28.21 ± 1.72	31.24 ± 0.45
Tot n-3	36.08 ± 2.94	27.92 ± 1.47
Tot n-6	8.67 ± 1.34	19.56 ± 0.51
DHA/EPA	1.81 ± 0.04	1.70 ± 0.05

**Table 3:** Astaxanthin (AST) yields (µg/g on a dry basis) obtained with different solvents—crude viscera oil (CVO), ethyl esters of total fatty acid obtained from CVO (TFA), polyunsaturated fatty acid ethyl esters enriched by short path distillation (SPD) (PUFAE), and exhausted fatty acid ethyl esters (EFA)—and different solvent extraction volume/matrix weight ratios (ER: 0.5; 1.0; 2.0), using WBP and DBP.

	ER	WBP	DBP	*p* < 0.05
CVO	0.5	80.21 ± 2.0 ^e^	52.56 ± 0.74 ^e^	*
TFA		86.14 ± 1.88 ^f^	47.81 ± 3.16 ^d^	*
PUFAE		31.78 ± 4.19 ^a^	31.89 ± 1.18 ^b^	-
EFA		27.17 ± 3.54 ^a^	19.02 ± 3.74 ^a^	*
CVO	1.0	97.99 ± 1.30 ^g^	64.22 ± 2.05 ^g^	*
TFA		105.23 ± 3.15 ^h^	58.41 ± 2.88 ^f^	*
PUFAE		38.83 ± 6.81 ^b^	38.96 ± 5.54 ^c^	-
EFA		33.20 ± 2.30 ^a,b^	23.23 ± 4.08 ^a^	*
CVO	2.0	149.06 ± 0.82 ^i^	97.68 ± 1.51 ^h^	*
TFA		160.06 ± 8.91 ^l^	88.85 ± 7.34 ^h^	*
PUFAE		59.06 ± 4.06 ^d^	59.26 ± 3.78 ^f,g^	-
EFA		50.50 ± 0.91 ^c^	35.34 ± 1.60 ^b,c^	*

Different lowercase letters in the same column indicate significant differences (a, b, c… *p* < 0.05). * in the same row indicates significant differences between wet and dry. Data are reported as mean ± standard deviation.

**Table 4:** Operating conditions used for degassing and molecular distillation.

	Degassing	Distillation
Flow (htz)	20	5
T (°C) Feed	40	40
T (°C) Condenser	25	60
T (°C) Residue	60	60
T (°C) Evaporator	80	80–240
Vacuum (mbar)	5	2 × 10^−3^
